# Traditional Japanese Kampo Medicine Yokukansan decreases phosphorylated tau and oligomeric tau

**DOI:** 10.1002/alz.093352

**Published:** 2025-01-09

**Authors:** Chika Mori, Ryota Yamamoto, Norimichi Shirafuji, Rei Asano, Hirohito Sasaki, Tomohisa Yamaguchi, Yuki Kitazaki, Yoshinori Endo, Soichi Enomoto, Tadanori Hamano

**Affiliations:** ^1^ Department of Neurology, University of Fukui, Matsuoka, Fukui Japan; ^2^ Department of Aging and Dementia (DAD), Faculty of Medical Sciences, University of Fukui, Fukui Japan

## Abstract

**Background:**

One of the pathological hallmarks in Alzheimer’s disease (AD) brain is neurofibrillary tangles (NFTs) composed of highly phosphorylated tau protein. Clinical benefit of traditional Japanese Kampo Yokukansan for dementia patients, including AD was suggested. In this study, we investigated whether yokukansan participates in the degradation of phosphorylated tau and toxic oligomeric species of tau by using cell culture model of tauopathy, M1C cells.

**Method:**

Neuronal cellular model of tauopathy, M1C cells which expresses wild type tau protein (4R0N) via tetracycline off induction (TetOff induction) was used in this study. M1C cells were treated with Ten to fifty ug/mL of Yokukansan and analyzed by Western blot analysis. Various phosphorylated tau antibodies (AT 180, AT270, and PS199/202) were used to detect phosphorylated tau. Oligomeric tau was examined by anti‐oligomeric tau specific antibody (TOC‐1) by dot blot analysis.

**Result:**

Up to 100 µg/mL of Yokukansan has no cytotoxic effects on M1C cells examined by morphological study, and ATP assay. WB analysis disclosed that of 10 to 50 µg/mL of yokukansan decreased phosphorylated tau detected by AT180, AT270, and PS199/202. Oligomeric tau detected by TOC1 also decreased by Yokukansan treatment. Major tau phosphatase GSK3β was inactivated by Yokukansan treatment. Autophagy marker P62 was downregulated by Yokukansan treatment.

**Conclusion:**

These results suggest that phosphorylated tau and oligomeric tau was reduced by Yokukansan via tau kinase inactivation and autophagy activation. Although the exact mechanisms of yokukansan on tau phosphorylation and oligomerization should be explored, these results shed light on the therapeutics of tauopathy, including AD.

